# Automated coding and selection of causes of death in Peru: a descriptive study, 2016-2019

**DOI:** 10.1590/S2237-96222023000300005.EN

**Published:** 2023-09-18

**Authors:** Javier Vargas-Herrera, Janet Miki, Liliana López Wong, Jorge Miranda Monzón, Rodolfo Villanueva

**Affiliations:** 1 Universidad Nacional Mayor de San Marcos, Unidad de Telesalud, Lima, Peru; 2 Vital Strategies, Programa de Registro Civil y Estadísticas Vitales, Lima, Peru; 3 Ministerio de Salud del Perú, Oficina General de Tecnologías de la Información, Lima, Peru; 4 Universidad Alas Peruanas, Escuela de Ingeniería de Sistemas e Informática, Lima, Peru

**Keywords:** Causes of Death, Mortality Records, International Classification of Diseases, Health Information Systems, Information Technology, Descriptive Epidemiology, Causas de Morte, Registros de Mortalidade, Classificação Internacional de Doenças, Sistemas de Informação em Saúde, Tecnologia da Informação, Epidemiologia Descritiva, Causas de Muerte, Registros de Mortalidad, Clasificación Internacional de Enfermedades, Sistemas de Información en Salud, Tecnologías de la Información, Epidemiología Descriptiva

## Abstract

**Objective:**

to describe software performance in the automatic selection of the underlying cause of death in Peru, between 2016 and 2019.

**Methods:**

this was a descriptive study on the software performance in the automated selection of the underlying cause of death over the years (chi-square test for trend) and the correlation between the type of death certificate and software performance (correlation coefficient and coefficient of determination).

**Results:**

a total of 446,217 death certificates were analyzed; the proportion of death certificates with the underlying cause of death increased from 69.6% in 2016 to 78.8% in 2019 (p-value < 0.001); it could be seen a direct linear correlation between electronic death certificates and software performance (correlation coefficient = 0.95; R^2^ = 0.89).

**Conclusion:**

the software showed good performance in the automatic selection of the underlying cause of death, with a significant increase between 2016 and 2019.

## Study contributions

**Main results:**

It could be seen good performance of the software for the automatic selection of the underlying cause of death, increasing from 69.6% in 2016 to 78.8% in 2019. There was a correlation between this result and the use of online death certificates by physicians.

**Implications for services:**

Automatic coding and selection of causes of death improve productivity and timeliness of information, contributing to the quality of the country’s information system.

**Perspectives:**

It is necessary to analyze the agreement between the medical terms in the software dictionaries used in South American countries in order to improve standardization and comparability of information on causes of death.

## Resumo

**Objetivo:**

descrever o desempenho de um *software* na seleção automática da causa básica de morte no Peru, entre 2016 e 2019.

**Métodos:**

estudo descritivo do desempenho de um *software* na seleção automatizada da causa básica de morte ao longo dos anos (teste qui-quadrado de tendência) e da correlação entre a forma de declaração de óbito e o desempenho do *software* (coeficientes de correlação e determinação).

**Resultados:**

foram analisadas 446.217 declarações de óbito; a proporção de declarações com causa básica de morte aumentou de 69,6%, em 2016, para 78,8%, em 2019 (p-valor < 0,001); observou-se correlação linear direta entre as declarações eletrônicas de óbito e o desempenho do *software* (coeficiente de correlação = 0,95; R^2^ = 0,89).

**Conclusão:**

o *software* apresentou bom desempenho na seleção automática da causa básica de morte, com aumento significativo entre 2016 e 2019.

## Resumen

**Objetivo:**

describir el desempeño de un software en la selección automática de la causa básica de muerte en Perú, entre 2016 y 2019.

**Métodos:**

estudio descriptivo de la tendencia del desempeño de un software para seleccionar la causa básica de muerte a través de los años (chi cuadrado de tendencia) y la correlación entre los certificados de defunción electrónicos y el desempeño del software (coeficientes de correlación y determinación).

**Resultados:**

se analizaron 446.217 certificados; la proporción de certificados con causa básica de muerte aumentó de 69,6% en 2016 a 78,8% en 2019 (p-valor < 0,001); se observó una correlación lineal directa entre certificados electrónicos y el desempeño del software (coeficiente de correlación = 0,95; R^2^ = 0,89).

**Conclusión:**

el software presentó un buen desempeño en la selección de la causa básica de muerte y aumentó significativamente entre 2016 y 2019.

## INTRODUCTION

Mortality information is useful for measuring the impact of health interventions.^1^ The World Health Organization (WHO) has established a set of guidelines and rules for the coding and selection of the underlying cause of death, aimed at medical certification; however, there are concerns about the reliability of this coding.^2^ ^)-(^ ^5^ Inter-coder agreement is higher in countries where specific coder engagement and retention policies have been implemented, reaching nearly 80%.^2^ ^),(^ ^6^ Similarly, automated coding software achieves comparable performance.

Incorrect use of coding and definition of the underlying cause of death affects data quality and its comparability between countries. There is software for automating these notifications, which is increasingly being adopted by countries,^7^ ^)-(^ ^10^ indicating a trend in the use of artificial intelligence in this process.^11^ In Latin America, there are experiences of implementing such software, from Mexico to Brazil, Chile, Colombia and Peru.^12^

Specifically, in Peru, in 2017, the Sistema Informático Nacional de Defunciones (SINADEF) was implemented, enabling death certificates to be performed via electronic forms in real time, improving the quality and timeliness of notifications.^13^ ^),(^ ^14^ The Peruvian Ministry of Health (MINSA) also decided to adopt the Iris software, developed by an international consortium led by the German Institute of Medical Information and Documentation, which uses an algorithm based on the rules of the International Statistical Classification of Diseases and Related Health Problems 10^th^ Revision (ICD-10), in order to automate the coding and selection of the underlying cause of death.^15^

The objective of this research note was to describe software performance in the automatic selection of the underlying cause of death in Peru between 2016 and 2019.

## METHODS

*Study design*

This was a descriptive study on software performance in the automatic selection of the underlying cause of death in Peru, between 2016 and 2019. This performance was defined as the software’s ability to obtain the underlying cause of death

*Setting*

By 2016, all deaths were documented on paper-based death certificates, transcribed into a desktop software called Vital Events and submitted to MINSA as files. In 2017, the Web-based SINADEF was implemented. This system allows death certificates to be registered in two ways: either directly typed online by physicians; or transcribed from paper-based formats. In 2018, the Iris software was adopted, and since 2016, mortality databases have been processed using this application to determine the underlying causes of death. The Iris dictionary was adapted with 12,246 medical terms in natural language, using the causes of death directly filled in by doctors as a reference.

*Participants*

This study included deaths that occurred in Peru between 2016 and 2019.^16^ Undeclared deaths and those that were not available at the time of data processing using the software were excluded.

*Variables*

The variables investigated were as follows: processed death certificate (with underlying cause of death; without underlying cause of death); recorded medical terms (with ICD-10 code; without ICD-10 code); type of error on the death certificate rejected by the software (syntax; code; system); type of death certificate (paper-based format; online); and year of death (2016 to 2019).

*Data sources and measurement*

The data source was comprised of death certificate databases covering the period from 2016 to 2019, provided by the MINSA in spreadsheet format. The data were processed using Iris on the following dates: 2016 mortality database on 06/01/2018; 2017 database on 04/26/2019; 2018 database on 6/20/2020; and 2019 database on 6/22/2021.

*Bias control*

Mortality database records underwent quality control to remove any potential duplicate records or modify records with inconsistent data.

*Statistical methods*

The variables obtained after processing using Iris were presented in simple frequency distribution tables. The trend analysis was performed using the chi-square test for trend. The Iris performance index (number of death certificates with underlying cause of death divided by the total number of death certificates) and the Iris performance index in ICD-10 coding (number of medical terms with ICD-10 codes divided by the total number of medical terms) were considered dependent variables; and the independent variable was the year of death. Pearson’s correlation coefficient and the coefficient of determination (R^2^) were used to analyze the linear correlation between the type of death certificate and Iris performance. The significance level used was 5%. Microsoft Excel® 2016 software was used for the analyses.

*Ethical aspects*

The study was based on the analysis of variables included in the mortality databases of the MINSA, also available on the National Open Data Platform https://www.datosabiertos.gob.pe/, which do not contain information that would allow the identification of deceased individuals.

## RESULTS

**Figure 1:**
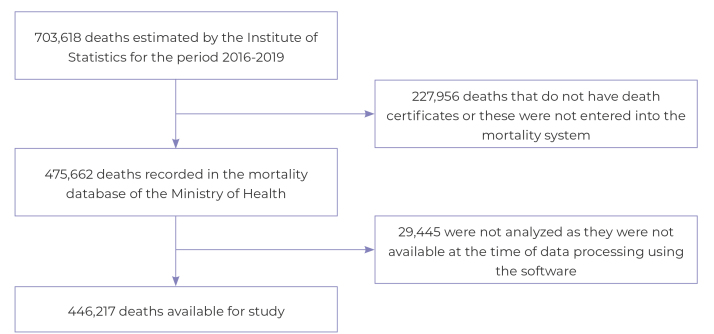
Selection process of studied deaths, Peru, 2016-2019.

Between 2016 and 2019, a total of 446,217 deaths of residents in all regions of Peru, recorded in the MINSA mortality database, were analyzed. This amount corresponded to 67% of the estimated deaths for the study period. Deaths that were not registered on the mortality system at the time of processing were excluded (Figure 1).

It could be seen a progressive increase in the software performance index, with the percentage of processed death certificates ranging from 69.6% in 2016 to 78.8% in 2019 (p-value < 0.001) (Table 1).

**Table 1:** Software performance in the selection of the underlying cause of death and death certificate processing (n = 446,217), Peru, 2016-2019

Software performance	2016		2017		2018		2019		p-value^a^
	N	%	N	%	N	%	N	%	
Processed death certificates									< 0.001
With underlying cause of death	67,697	69.6	86,848	71.8	79,979	74.8	95,405	78.8	
Without underlying cause of death	29,542	30.4	34,174	28.2	26,926	25.2	24,653	21.2	
Total	97,241	100.0	121,024	100.0	106,905	100.0	121,047	100.0	
Processed medical terms									< 0.001
With ICD-10 codes	222,446	87.2	321,904	89.8	299,988	91.1	346,635	92.7	
Without ICD-10 codes	32,641	12.8	36,623	10.2	29,401	8.9	27,297	7.3	
Total	255,087	100.0	358,527	100.0	329,389	100.0	373,932	100.0	
Death certificates rejected by type of error									< 0.001
Syntax error	-	-	744	2.3	545	2.1	582	2.4	
Code error	26,819	90.8	30,337	94.0	24,616	94.9	23,130	93.8	
System error	2,725	9.2	1,185	3.7	790	3.0	941	3.8	
Total	29,544	100.0	32,266	100.0	25,951	100.0	24,653	100.0	
Type of death certificate									< 0.001
Paper-based format	96,605	100.0	85,986	71.0	32,227	30.1	25,397	21.0	
Online	636	0.0	35,038	29.0	74,678	69.9	95,650	79.0	
Total	97,241	100.0	121,024	100.0	106,905	100.0	121,047	100.0	

a) Chi-square test for trend.

There was also an increasing trend in the software performance index in ICD-10 coding, with a progressive increase in the proportion of this performance: from 87.2% in 2016 to 92.7% in 2019 (p-value < 0.001). However, the highest proportion of errors in the records that the software failed to process were coding errors (Table 1).

**Figure 2:**
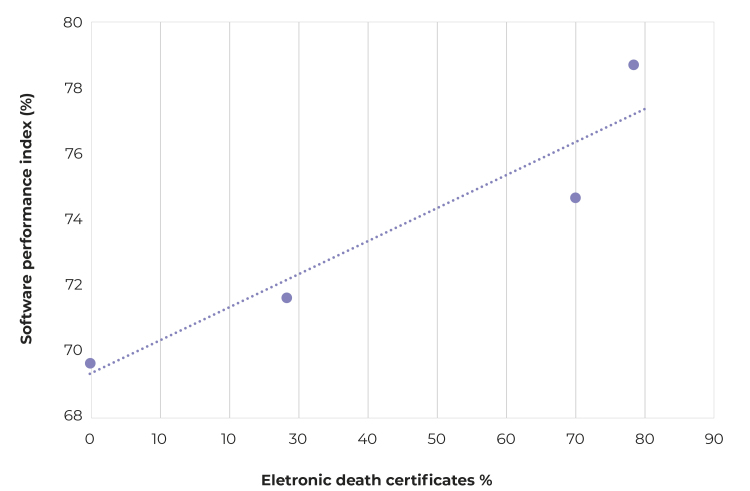
Correlation between the proportion of death certificates produced in electronic format and the software performance index in the selection of the underlying cause of death, Peru, 2016-2019

Notes: Pearson’s linear correlation = 0.95; Coefficient of determination R^2^ = 0.89.

It could be seen a direct linear correlation between the proportion of death certificates directly filled out by physicians on SINADEF and the Iris performance: Pearson’s correlation coefficient = 0.95; R^2^ = 0.89 (Figure 2).

## DISCUSSION

During the study period, the software performance increased due to the progress in its ability to code the terms used by physicians for reporting causes of death with ICD-10. There was a correlation between the proportion of declarations directly filled out by the physicians and the software performance. The implementation of SINADEF played an important role in this process, enabling the development of a dictionary adapted to the Peruvian context and contributing to an increasing number of medical terms each year. Studies have shown improvements achieved after training physicians in filling out death certificates, and in the quality of the data they record,^17^ ^),(^ ^18^ in addition to the possibility of improving the software performance.

One limitation of this study lies in the lack of an analysis of the agreement between the software and the application of rules by experienced coders. In Peru, this type of analysis is difficult because, before the implementation of SINADEF, most death certificates were coded by employees without formal training in ICD-10. A second limitation is related to the fact that, in this study, the Iris performance was not analyzed by sociodemographic variables or disease group.

The highest performance of the software was around 80%. A study conducted in São Paulo, in 2010, with a sample of 666 deaths aimed at testing the software Portuguese dictionary, found a performance of 95%.^19^ In the Netherlands, during a study on the implementation of an automated coding system with data from 134,262 deaths that occurred in 2009, there was an increase in performance from 17% in the first batch to 69% in the last batch, after a series of improvements in the dictionary.^20^ In Spain, a study to assess the impact of automating cause of death records on mortality in the autonomous community (geopolitical macro-region) of Navarra, based on 5,060 deaths that occurred in 2014, identified a performance of 90%.^21^ When evaluating the use of Iris in a small sample of deaths in Burkina Faso, a performance of 90% was found.^10^

In this study, automatic coding of medical terms for causes of death was 93%, while in Italy, in 2016, this proportion was 78%.^22^

Most of the errors that led the software to reject a death certificate occurred during coding: typos, spelling errors, or errors with unusual characters.^19^ However, the software also faces challenges in accurately coding external causes of death, because forensic medical examiner use a wide range of causes of death, which affects the efficiency of the dictionary.^20^

There is a global movement towards automated selection of the underlying cause of death. Nearly all countries in the European Union use Iris. In Latin America, the software is being implemented in several countries. In Brazil, it has been integrated into a mobile application for doctors, aiming to improve the completion of the Death Certificate (DC).^23^ ^),(^ ^24^

It can be concluded that there is a trend of improvement in the performance of the software for selecting the underlying cause of death in Peru. This improvement seems to be associated with the implementation of the SINADEF and the optimization of the dictionary of medical terms. Further studies on Iris are needed to assess the impact of the software on mortality statistics. Taking into consideration that its implementation in the region will enhance data comparability, it is necessary to study the agreement between the medical terms of the dictionaries used in South American countries.

## References

[1] Suthar AB, Khalifa A, Yin S, Wenz K, Ma Fat D, Mills SL (2019). Evaluation of approaches to strengthen civil registration and vital statistics systems: a systematic review and synthesis of policies in 25 countries. PLoS Med.

[2] Antini C, Rajs D, Muñoz-Quezada MT, Mondaca BAL, Heiss G (2015). Reliability of cause of death coding: an international comparison. Cad Saude Publica.

[3] Минаева А, Вайсман К (2015). The peculiarities of coding and the determination of the primary cause of death from the diseases induced by the human immunodeficiency virus in accordance with ICD-10. Sud Med Ekspert.

[4] Winkler V, Ott JJ, Becher H (2010). Reliability of coding causes of death with ICD-10 in Germany. Int J Public Health.

[5] Gamage USH, Adair T, Mikkelsen L, Mahesh PKB, Hart J, Chowdhury H (2021). The impact of errors in medical certification on the accuracy of the underlying cause of death. PLoS One.

[6] Harteloh P, Bruin K, Kardaun J (2010). The reliability of cause-of-death coding in The Netherlands. Eur J Epidemiol.

[7] Eckert O (2019). Electronic coding of death certificates. Bundesgesundheitsblatt Gesundheitsforschung Gesundheitsschutz.

[8] Rey G (2016). Death certificate data in France: production process and main types of analyses. Rev Med Interne.

[9] Barro SG, Rey G, Staccin P (2019). Study of the usability of an automated coding software for causes of death in an African context. Stud Health Technol Inform.

[10] Rey G, Bounebache K, Rondet C (2018). Causes of deaths data, linkages and big data perspectives. J Forensic Leg Med.

[11] Falissard L, Morgand C, Roussel S, Imbaud C, Ghosn W, Bounebache K (2020). A deep artificial neural network-based model for prediction of underlying cause of death from death certificates: algorithm development and validation. JMIR Med Inform.

[12] Pan American Health Organization (2015). istema de Codificación Automatizada de causa de muerte “Iris”.

[13] Vargas-Herrera J, Ruiz KP, Nuñez GG, Ohno JM, Pérez-Lu JE, Huarcaya WV (2018). Resultados preliminares del fortalecimiento del sistema informático nacional de defuncionesev. Peru Med Exp Salud Publica.

[14] Vargas-Herrera J, Monzón MJ, Wong LL, Ohno JM (2022). La cobertura de muertes con certificación médica en el Perú, 2012-2019. An Fac med.

[15] Iris Institute (2022). Iris user reference manual V5.8.1S2.

[16] Instituto Nacional de Estadística e Informática (2019). Boletín Especial nº 24 - Perú: estimaciones y proyecciones de la población nacional, por año calendario y edad simple, 1950-2050.

[17] Miki J, Rampatige R, Richards N, Adair T, Cortez-Escalante J, Vargas-Herrera J (2018). Saving lives through certifying deaths: assessing the impact of two interventions to improve cause of death data in Perú. BMC Public Health.

[18] Vargas-Herrera J, Meneses G, Cortez-Escalante J (2022). Physicians’ perceptions as predictors of the future use of the national death information system in Peru: cross-sectional study. J Med Internet Res.

[19] Martins RC, Buchalla CM (2015). Automatic coding and selection of causes of death: an adaptation of Iris software for using in Brazil. Rev Bras Epidemiol.

[20] Harteloh P (2020). The implementation of an automated coding system for cause-of-death statistics. Inform Health Soc Care.

[21] Floristán YF, Osinaga JD, Prieto JC, Perez JA, Moreno-Iribas C (2016). Coding Causes of Death with IRIS Software. Impact in Navarre Mortality Statistic. Rev Esp Salud Publica.

[22] Orsi C, Navarra S, Frova L, Grande E, Marchetti S, Pappagallo M (2019). Impact of the implementation of ICD-10 2016 version and Iris software on mortality statistics in Italy. Epidemiol Prev.

[23] Suárez LC (2018). Primer bienio de estadísticas de mortalidad con el codicador automático Iris de causas de muerte. Gac Sanit.

[24] Ishitani LH, Cunha CCD, Ladeira RM, Corrêa PRL, Santos MRD, Rego MAS (2019). Evaluation of a smartphone application to improve medical certification of the cause of death. Rev Bras Epidemiol.

